# Distinction Between Dysplasia, Malformation, and Deformity—Towards the Proper Diagnosis and Treatment of Hip Development Disorders

**DOI:** 10.3390/diagnostics15121547

**Published:** 2025-06-18

**Authors:** Jacek Dygut, Monika Piwowar

**Affiliations:** 1 Medicare Clinic, Brook House, Lime Tree Court, Cardiff Gate Business Park, Mulberry Dr, Cardiff CF23 8AB, UK; jacekdygut@gmail.com; 2 Department of Bioinformatics and Telemedicine, Medical College, Jagiellonian University, 31-034 Kraków, Poland

**Keywords:** dysplasia malformation, deformity, DDH, hip disorder

## Abstract

(1) Background: This publication focuses on processes that disrupt the proper development of the hip. Four pathomechanisms underlying human developmental defects are described in the literature, i.e., dysplasia, malformation, disruption, and deformity. In the case of hip development, arguably the greatest challenge involves confusion between dysplasia and deformity, which often leads to misdiagnosis, incorrect nomenclature, and incorrectly chosen treatment. (2) Methods: A review of the scientific literature was performed. (3) Results: The paper presents a description of hip joint development disorders in the context of their pathomechanisms. An attempt was made to answer the question of whether these disorders are rooted in a primary disorder of tissue growth, resulting in incorrect anatomy, or are the result of anatomical deformations with secondary modifications in tissue structures—of a degenerative or adaptive nature—based on Delpech–Hueter–Volkmann growth and remodeling laws. In addition, the emphasis is placed on the presence of so-called clinically and diagnostically mute cases. We suggest augmenting diagnostic procedures with genetic tests to increase the sensitivity of screening. (4) Conclusions: Based on the arguments, a new division of developmental hip disorders is proposed.

## 1. Introduction

This publication aims to draw attention to the extent to which developmental disorders of the hip are based on the pathomechanism of dysplasia, and to what extent they are based on the pathomechanism of deformity, as well as to refine diagnostic schemes to distinguish these disease entities, which are currently not fully refined despite the technical possibilities.

Many studies of hip development disorders have been performed [1], leading to many proposed diagnostic and treatment options [2]. Despite the impressive collection of studies documented in scientific publications, the etiology of developmental disorders of the hip joint still cannot be formulated with clarity [1,3,4,5]. Even so, the etiology of these diseases is known to be multifactorial, combining genetic factors (with varying intensity and expression periods) and environmental factors affecting the fetal and postnatal period [6,7]. Normal hip growth and development depend on a genetically determined balance between the growth of the acetabular and triradiate cartilages and a properly located and cantered femoral head [6,8,9].

Experimental studies have revealed that the development of the acetabulum depends on a coded geometric pattern [10,11]. The concave shape of the acetabulum results from the presence of a round femoral head inside. In addition, many other factors affect the acetabular depth, including the growth within the acetabular cartilage, growth through apposition under the perichondrium layer, and the growth of adjacent bones (iliac, sciatic, and pubic) [8,9].

The incidence of developmental hip growth disorders varies by population. It is close to 0% among newborns in China and Africa, but rises to 1% in Caucasian newborns, with the incidence of hip dislocations at approximately 0.1% [12]. Notably, these differences may result from environmental factors, such as the manner of childcare, rather than genetic issues. A positive family history of hip development disorders is found in 12–33% of patients and is more often observed in female children (80% of cases) [12].

As described in the literature, three independent but equivalent pathomechanisms underlying hip joint development disorders—i.e., malformation, dysplasia, and deformity—can be identified [4]. The fourth pathomechanism of developmental disorders, disruption, has been excluded from further consideration because its relevance to developmental hip disorders remains unproven. Except for deformity, which falls within the group of “packaging problems”, the three remaining pathomechanisms are collectively referred to as “production problems” [12].

Many recent scientific reports do not take into account the differences between the above-mentioned pathomechanisms. They are treated as synonymous, usually grouped under one name, i.e., developmental dysplasia of the hip (DDH) [13,14,15]. Some publications use the following terms interchangeably: congenital hip dysplasia [3], congenital hip dislocation [16], and developmental deformity of the hip [17]. Others contain statements such as “… malformation of anatomical structures occurs in dysplasia, which at the time of embryonic development were still normal …” or “… developmental hip dysplasia is more deformity than malformation …” [12]. According to the International Classification of Diseases and Health Problems (Q65 to Q79), the term “congenital hip dislocation” remains valid [18].

To precisely explain the need to distinguish and organize these issues, we present and explain the following definitions, based on which a new division of hip developmental disorders is proposed.

**A congenital defect** is a disorder present since birth. It is a general term, broadly describing the structural, behavioral, functional, and metabolic damage that occurs in prenatal life, and which is diagnosed after birth or later in life [19].

**Malformation** (Latin: *malformatio*) is a term frequently used by English-speaking authors to refer to developmental disorders in general. More specifically, however, malformation represents just one of the four pathomechanisms of developmental disorders [4]. Malformation concerns developmental disorders, but only during the embryonic period. Referring to developmental changes that occur after this period as “malformation” is incorrect.

Malformation of the mesenchymal primordium of the hip joint is a type of birth defect caused by a primary disorder of hip development during the embryonic period, during differentiation, or during organogenesis. The primary disorder affects cell proliferation, differentiation, migration, apoptosis, and/or cell intercommunication processes. The primary impairment of cell function inhibits, delays, or directs tissue development in the wrong direction, causing the improper formation of anatomical structures of the hip [4]. Malformation underlies the development of congenital teratogenic hip dislocation. This is in line with Tachdjian’s opinion on malformation: “It is important to differentiate deformations from malformations. (…) Malformations cannot be corrected directly, whereas deformations can often be revised relatively easily either by elimination of the deforming force or by counteracting the force with stretching, casting, or bracing”.

It is a mistake to use the terms deformation (or deformity) and malformation as synonyms. Nevertheless, they are still used interchangeably. Suffice it to mention that the term malformation inaccurately describes various issues; for example, the following:

- malformation as a synonym of distortion, which, often for this reason, is incorrectly called deformity (deformation)—another pathomechanism of developmental defect that is completely different from malformation.
- malformation as a developmental defect, confusing the interpretation of medical publications.

**Dysplasia of the hip** is a type of disorder in which abnormally developing tissue (often excessively flaccid) results in faulty hip anatomy and then evolves. Anatomical structures of the hip, normal during embryonic development, gradually become abnormal for various reasons [10,12,20]. Dysplasia may be environmentally or genetically conditioned. Dysplastic changes, along with malformation and disruption, may be collectively referred to as “production problems”. Hip dysplasia can occur both in the prenatal (early dysplasia) and postnatal (late dysplasia) period [21]. These disorders do not tend to self-heal [12].

**Deformity of the hip** joint is an example of a developmental disorder in which properly developed structures are deformed during growth as a result of mechanical factors [10]. This can occur both in the prenatal and postnatal periods. If the mechanical factor is active in the prenatal period, then we may refer to it as a “packaging problem” associated with intrauterine fetal modeling. Disorders of this type are unlikely to cause growth disorders—rather, they tend to disappear with age and self-heal [12].

**Figure 1:**
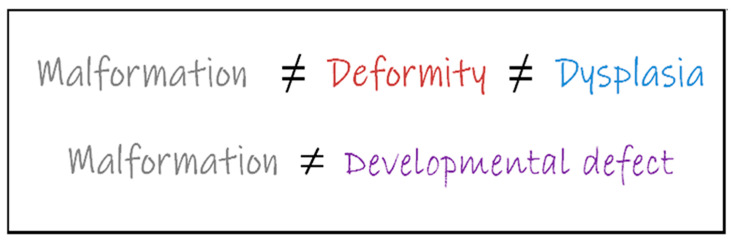
Terms used in the context of hip disorders. Due to different pathomechanisms, the concepts presented in the figure are not equivalent.

**Distortion** is a pathological condition in which the shape of a part of the body has remodeled beyond the normal range. Developmental distortion of the hip is an overarching term that describes both deformity and malformation, as well as dysplasia. In connection with the above, deformity in medical terms can by no means be dysplasia, let alone malformation (Latin *malformatio*), but all of the above can lead to developmental distortions of parts of the human body [12] (Figure 1).

**A developmental disorder** is a disorganization in the anatomical structure of the osteoarticular system that appears after some time, is absent or invisible at birth, and has a tendency to either self-heal or worsen over time. In English literature, the term “structural defect” is often used in the context of developmental disorders to emphasize the anatomical nature of the defect.

When referring to dysplasia and deformity, it is reasonable to introduce an additional term—“developmental disorder”—because we are talking about anomalies with a tendency to self-heal or become more severe over time [4]. Developmental dysplasia or a developmental deformity of the hip, depending on the time of occurrence, can be early (primary—invisible and present at birth) or late (secondary—absent and appearing after some time) [12,22].

As disruption has not been described in the context of developmental hip joint disorders, and malformation is relatively easily diagnosed as a component of congenital anomalies, the main focus is on the distinction between dysplasia and deformity.

The aforementioned distinction is extremely important because it projects the course of treatment and prognosis of hip joint development disorders. It influences the choice of various conservative or surgical treatment strategies aimed at maintaining or restoring the normal growth potential of anatomical structures [12]. Misdiagnosis can lead to incorrect therapeutic management and, consequently, to deepening disability, thus significantly increasing the cost of treatment [23,24].

The following is a discussion of the pathogenesis of developmental hip disorders, with a focus on dysplasia and deformities.

## 2. Materials and Methods

A review of the literature on dysplasia, malformations, and deformities in the context of developmental disorders of the hip joint was performed. The analysis of scientific studies was based on the definitions of the above terms. Then, a diagram of the developmental disorders of the hip was constructed. The literature review was conducted based on data deposited in the PubMed database, covering the period up to 2025, and in books in the field of pediatric orthopedics. The focus was only on articles containing issues related to developmental disorders of the hip joint (dysplasia, deformity) and genetic disorders of a teratogenic nature (malformation).

## 3. Results and Discussion

### 3.1. Malformation as a Disorder of the Hip Joint Formation Process

The pathomechanism of malformation cannot be the root cause of a developmental hip disorder leading to dysplasia because it is only in the seventh week of life within the mesenchyme that the hip joint develops a fissure, secreting the future femoral head and the acetabulum. Therefore, the first period when hip dislocation, and thus developmental hip dysplasia, may occur is the eleventh week of fetal life—the time when the hip joint is fully formed [6]. In the case of malformation, the most frequent causative factor is congenital anomalies syndrome, which generally does not pose major diagnostic difficulties. The effects of such congenital changes are present and visible immediately after childbirth [25].

### 3.2. Dysplasia and Deformity as Developmental Disorders of the Hip Joint

**Figure 2:**
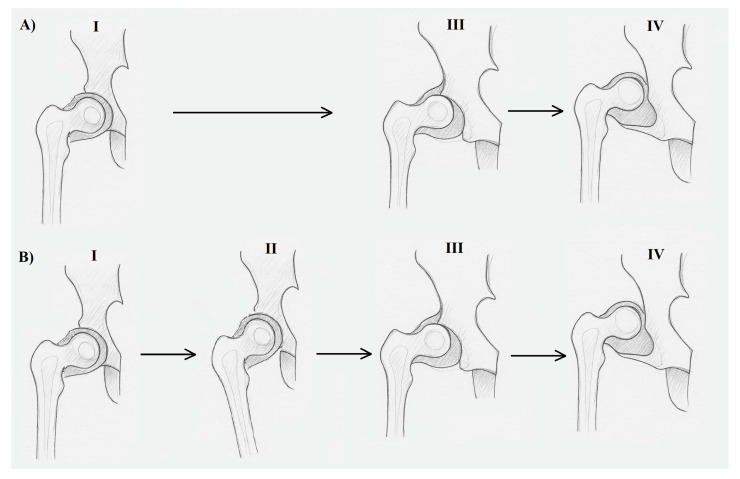
The difference between dysplasia (**A**) and deformity (**B**) in developmental disorders of the human hip joint. (**A**) I—properly formed hip joint after the embryonic period, in the fetal period; (**A**) III—subluxation of the hip joint due to genetically based dysplastically changed tissues forming the joint; (**A**) IV—developmental dislocation of the hip joint due to tissue dysplasia; (**B**) I—properly formed hip joint after the embryonic period; (**B**) II—adduction of the hip joint due to a mechanical factor (ultra positions in the fetal period or postnatal adduction contracture); (**B**) III—subluxation of the hip joint with a normal tissue structure due to the action of a mechanical factor; (**B**) IV—developmental dislocation of the hip joint due to the action of a mechanical factor. Stages III and IV in both dysplasia and deformity are extremely difficult to distinguish in both clinical and histopathological examinations, although some come from primary changes (dysplasia) and others from secondary changes (deformity).

In the literature, dysplasia is considered via two aspects: dysplasia as a precancerous lesion (applies only to epithelial tissue, which is outside the scope of this discussion), and dysplasia as a developmental disorder, involving incorrect organization or function of cells in a specific tissue (described as “production problems”), which is under consideration in this study [12]. Dysplasia, which is a developmental disorder of the hip, can be grouped under the so-called **osteoarticular dysplasia epiphyseal type** [20,26]. It is characterized by the abnormal growth potential of tissue structures underlying anatomical and functional changes in the growing hip [8,9]. In such cases, using the term **dysplasia** is fully justified (Figure 2A).

Risk factors for developmental dysplasia can include environmental or genetic conditions on both the mother’s and child’s side. Many scientific reports contain information on the impact of elevated levels of biochemical factors on the occurrence of developmental dysplasia, e.g., female hormones (e.g., relaxin, estrogens) and biochemical markers of nutritional status (e.g., calcium, vitamins C and D) [1]. Regarding the relationship between the concentration of the hormone relaxin, derived from the mother, in the blood of the fetus and the instability of hip joints, it was established that facts contradict the earlier assumption that hip instability coincides with increased relaxin concentrations in newborns. Instead, results indicate that hip instability frequently accompanies decreasing relaxin levels. The authors, therefore, assumed poorer mobilization of the pelvis and the birth canal during pregnancy as a result of a lower concentration of relaxin, which may result in greater pressure on the fetus in the perinatal phase [27]. Abnormalities caused by the disturbed balance of biochemical factors on the part of the mother can be expressed in the immaturity of the tissues of the child’s hip joint and the delay of their development in the prenatal period [27,28,29]. Such changes may be temporary and transient. With the right positioning of the hips, proper care of the newborn, and then the baby, in most cases, the correct architecture of the anatomical elements of the hip can be restored [7].

Other studies report genetic disorders of the fetus underlying dysplastic changes in the hip joints. It has been confirmed that relaxation of ligaments and joint capsules, as well as irregularities in collagen metabolism, are associated with developmental dysplasia. Some genes have been linked to dysplasia of the hip in small studies, including *COL1A1*, *MMP13*, *IL-6*, *ADAMTS4*, *FRZB*, *CX3CR1*, *ASPN*, *DKK1*, *PDRG1*, *GDF5*, *UQCC1*, *TGF-β1*, *TBX4*, *CX3CR1*, *HOXD9*, and *PAPPA2*. Some genes are involved in connective tissue development, skeletal development, and joint formation, as well as being linked to limb development and joint positioning [30,31]. Other studies have identified a significant downregulation of genes in the ferroptosis signaling pathway. Thus, the ferroptosis signaling pathway may be associated with the pathogenic mechanism of DDH [32]. However, none of these genes are currently used in routine clinical testing due to small sample sizes, lack of replication across populations, and their unclear predictive value. It is still in early stages—in other words, not yet clinically actionable.

It has also been shown that some types of HLA A, B, and D—as well as mutations in specific genes or regulatory sequences, including genetic changes on chromosome 17 (17q21)—predispose the child to developmental hip disorders of a dysplastic nature [5,33,34]. This group likely covers cases of developmental disorders characterized by the prevalence of residual, recurrent, and late forms that are resistant to treatment.

In situations (as assumed by the Delpech law) where a correctly growing hip joint is affected by an external mechanical force, whether intracorporeal (e.g., extra-articular contracture) or extracorporeal (e.g., incorrect position of the lower limb), leading to deformation of its anatomical structure, we are dealing with deformity [12]. The prolonged action of such factors, combined with ongoing growth, may result in ultimate subluxation or even full dislocation. In such cases, the term “deformity” is fully justified (Figure 2B).

In deformity, a change in the shape of the growing hip joint due to external extra-articular forces is not accompanied by disruption of the structure and tissue function in the initial phase, as is the case with dysplasia. Uneven distribution of forces acting on the roof of the growing acetabulum by the moving head leads to inhibition of the growth of cartilage and bone tissue, their sclerosis, and ultimately steep positioning of the acetabulum roof [10]. Atrophy of the acetabulum roof is accompanied by excessive bone growth within its fossa, i.e., in the unloaded zone. As a result of the loss of modeling and sliding out of the femoral head during acetabulum growth, the acetabulum bottom becomes bold, the acetabulum roof flattens, and the acetabulum becomes shallow.

These processes of growth and remodeling of cartilage and bone tissue are secondary and comply with the Wolff–Delpech laws, later developed by Hueter–Volkmann, Pauwels, and Arndt-Schmidt [12].

The Hueter–Volkmann law also explains the presence of the most frequently observed pathological change in early postnatal hip dislocation, which is neolimbus (Ortolani positive symptom) [6,12]. The lack of physiological interaction (mutual pressure) between the head and the posterior edge of the acetabulum leads to excessive hypertrophy of the hyaline cartilage (neolimbus) in the upper, posterior, and lower periphery of the acetabulum, with the labrum curved out (pulled by a joint capsule in the dislocated hip) [12].

Deformity caused by mechanical factors, which is usually the result of intrauterine modeling, especially in the last trimester of pregnancy (hence the term “packaging problems”) usually does not cause disturbances in the growth potential of joint tissues, as observed in dysplasia or malformation. Instead, it tends to self-heal and rarely leads to relapse [12].

This group includes cases of fetuses with an abnormal breech position and an ultra position of limbs that self-heal or recover in the postnatal period, assisted by short-term conservative therapy [7]. Treatment involving the restoration of the compact joint with concentric maintenance of the head in the acetabulum ensures optimal development conditions. If repositioning is effectively maintained, the acetabulum, femoral head, and femoral neck in anteversion undergo remodeling as a result of their normal growth potential. Restoration of the correct and stable joint connection between the femoral head and the acetabulum can lead to remodeling of the deformity and normalization of the morphology of the hip (due to developmental plasticity) [12]. The potential and growth time of the hip joint are closely related and depend on genetic and environmental factors [12]. These include genetic variations (e.g., of the SNP type) modulating the activity of proteins important from the point of view of tissue function, along with factors such as nutrition, general health, hormone concentration, mechanical forces, and physiological age [34]. Therefore, during diagnostics and treatment, dysplastic and deformative cases should be considered together.

Time can be an ally or an enemy, depending on whether growth potential remains normal. If it does, as in the case of deformity, then growth may promote the development of correct anatomical structures (after correction and concentric arrangement of the elements of the hip joint). In the absence of normal growth potential, as with dysplasia, the deformity of anatomical structures may deepen [12] as growth progresses, even if a concentric position of the hip is achieved (recurrent, residual dysplasia resistant to treatment). Joint space narrowing can be an early radiological indicator of hip osteoarthritis in the context of dysplasia [35]. Such difficult-to-treat dysplasia of the hip joint may require corrective surgical treatment (change of acetabular inclination, change of the cervical-shaft angle and the antetorsion angle) after the person has completed growth.

### 3.3. Clinically and Diagnostically Mute Hip Developmental Disorders

It is nearly impossible to distinguish between dysplasia and hip joint deformity based on physical examination and imaging, e.g., ultrasound, X-ray, and MRI. The procedures used in many countries, which call for imaging when Ortolani, Barlow, and limited abduction tests are positive, may not be sufficient [12,15]. Because of the difficulty in correctly distinguishing between these two pathomechanisms, misdiagnosis often follows, and terminology is incorrectly applied. In the diagnosis and treatment of developmental hip joint disorders, there is a notable lack of uniform diagnostic and therapeutic standards in various countries around the world. This applies to the frequency of examinations and their complementarity [12,15,36,37,38]. There is also the danger of not detecting so-called clinically mute developmental disorders [6,39]. These are cases in which physical examination does not provide information about pathological changes at the joint level, which, however, become evident under imaging [15]. The literature describes cases of otherwise healthy children with normal physical examinations and radiographs of the hip in the first 3 months of life, who later developed hip dislocations [21].

**Figure 3:**
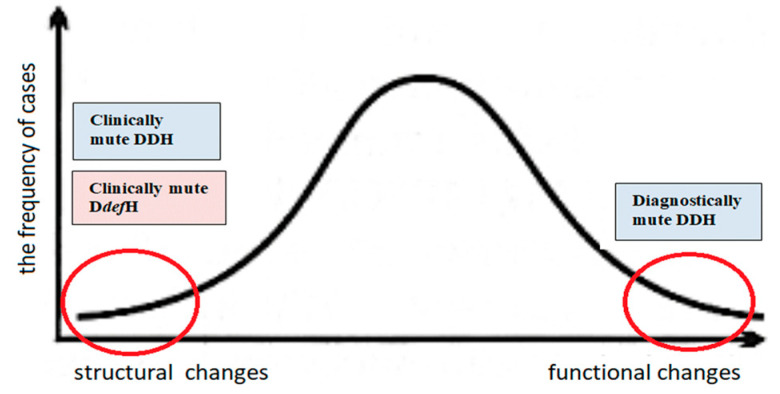
Schematic representation of the incidence of developmental hip disorders. Cases that are undetectable (mute) by physical examination or additional examinations are marked with red circles at both ends of the curve. Clinically mute developmental disorders—cases in which physical examination does not provide information about pathological changes at the joint level, which, however, become evident under imaging. Diagnostically mute developmental disorders—cases when physical examination clearly indicates a developmental disorder of the hip joint, and even its total displacement, while additional imaging tests do not confirm such abnormalities.

The opposite situation—involving diagnostically mute developmental disorders—may also occur. This condition occurs when physical examination clearly indicates a developmental disorder of the hip joint, and even its total displacement, while additional imaging tests do not confirm such abnormalities [21]. The joint can be anatomically normal but functionally abnormal, e.g., due to the limitation of hip abduction. Passivity towards an asymmetrical movement of the hip abduction can lead to a developmental hip disorder, which often results in dislocation [15]. The occurrence of the above-mentioned cases is not frequent, but doctors, orthopedists, pediatricians, and family doctors should be aware that they occur (Figure 3). In this context, it should be noted that, in cases of developmental hip dysplasia and deformity disorders, physical examination remains the priority, but it must be supplemented with imaging tests. The complementarity of physical examinations and imaging tests may reduce the number of undetected diagnostically and clinically silent cases.

The most common are cases in which structural changes are accompanied by functional disorders (peak on the curve). Cases undetectable (mute) by physical examination or additional examinations are marked with red circles at both ends of the curve.

### 3.4. Early Detection of Cases of Late Dysplasia

In many cases of dysplasia in the first weeks of the child’s life, the doctor will not observe pathological anatomical changes of the hip and will not detect tissue changes even though they are present. These changes may lead to joint discongruity, which may deepen during hip development, as observed in late forms of dysplasia.

In the case of late dysplasia, diagnoses are most often made in a situation when there are degenerative changes in the hip joint presenting with pain in the 3rd or 4th decade of life [6].

Treatment implemented at this stage is symptomatic and less effective than it might have been had the problem been noticed earlier. To avoid such situations, the possibility of implementing additional diagnostic tests should be considered. Given the development of high-throughput methods and the corresponding decrease in their cost, it is worth considering the implementation of a genetic testing procedure for detecting genetic variations responsible for pathological phenotypes. Knowledge about the genotype of patients with dysplastic changes would facilitate therapeutic planning in early cases of dysplasia, particularly concerning abnormalities that are not phenotypically revealed until a later period in the patient’s life [15]. Such variations may explain the presence of short- and long-term tissue function disturbances, which lead to anatomical disorders of acetabulum development along with functional disorders of extra-articular tissues (contracture, excessive flaccidity) [6,40,41].

Several papers have been published on the topic of the genetic background of hip dysplasia; however, these studies always focus on changes occurring in specific genes. A study of the full range of genomic sequences with all potential variations (NGS sequencing studies) would allow us to describe the entire spectrum of variations comprising the observed dysplastic changes [33,34]. Identification of, e.g., single nucleotide changes (correlated with pathological phenotypes and affecting regulatory sequences that modulate protein functions) could significantly increase the level of diagnostic accuracy [42,43].

Considering the above facts, it should be noted that, nowadays, there may still be clinically and diagnostically mute cases in neonatal hip tests, since genetic diagnostics are not yet routine. Integration of genetic testing with medical procedures would enable the objective assessment of the situation at an early stage in both early and late dysplasia.

Detection of differences at the level of genomic DNA, characteristic of the group of patients with dysplasia, would allow the classification of this disorder with varying degrees of aggressiveness based on the obtained genetic profiles. In the future, this could serve as a tool for planning personalized therapy and become part of international standards [12,15,36,37,38]. Incorrect diagnosis resulting from incomplete patient data is the main cause of disability in childhood and adulthood, and treating such disability imposes a serious burden on state budgets [23]. With modern diagnostic tools, the consequences of overlooking the defects could be largely avoided. In addition, this would sensitize the attending physicians to the possibility of late presentation of the abnormality, its resistance to treatment, and eventual recurrence. Vigilance in the treatment process would facilitate thoughtful planning of the course of therapeutic management and thus improve the quality of life of patients in the future [24].

### 3.5. Classification of Developmental Hip Disorders

**Figure 4:**
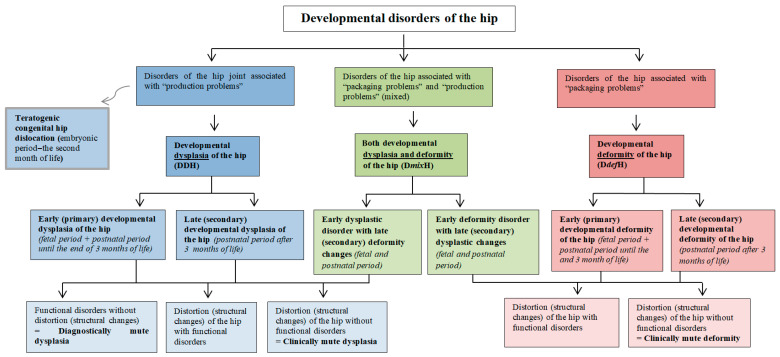
Proposed division of developmental disorders of the hip joint. The diagram differentiates pathophysiological mechanisms, i.e., dysplasia and deformity, leading to various disorders of hip joint development.

Based on the above considerations, a classification of developmental hip disorders was proposed, depending on the etiology of the defect (Figure 4).

**I.**  **Developmental disorders of the hip joint associated with “production problems”**

In these abnormalities, the tissues forming the hip joint are primarily defective (dysplastic). Primary disturbed tissue development results in secondary disturbed hip joint anatomy.

**(1)**  **Teratogenic congenital hip dislocation**

This is an example of a “tissue production” disorder that involves malformation at the embryonic stage, i.e., before the end of hip joint differentiation. It can be caused by genetic and/or biochemical and/or biophysical factors in the embryonic period.

**(2)**  **Developmental dysplasia of the hip (DDH)**

Another “tissue production” disorder involves dysplasia, which may begin to manifest in the prenatal (fetal) period, i.e., from the time of hip joint formation (at 11–12 weeks of age) throughout the postnatal period. It may be caused by genetic and/or environmental factors.

Depending on the time of onset, DDH can be divided into:

 **(A)** **Early (primary) developmental dysplasia of the hip**

This disorder refers to the fetal phase of the prenatal period, including the postnatal period up until the end of the 3rd month of life.

 **(B)** **Late (secondary) developmental hip dysplasia**

This disorder refers to the postnatal period—after 3 months of age.

**II.**  **Developmental disorders of the hip associated with “packaging problems”**

In these disorders, properly formed tissues of the anatomical structures of the hip joint are deformed due to the prolonged presence of mechanical factors. An untreated deformity may, in the long term, cause secondary hip tissue changes following the remodeling and adaptation laws of Delpech–Hueter–Volkmann—this mainly concerns bone tissue (atrophy and sclerosis in the overloaded zone, hypertrophy, and low-density bone structure in the unloaded zone) [22,44].

**(1)**  **Developmental deformity of the hip (D*def*H)**
  **(A)**  **Early (primary) developmental deformity of the hip**

This disorder occurs in the prenatal (fetal) period and the postnatal period until the end of the 3rd month of life. Depending on the time of exposure to the deforming mechanical factor, we can distinguish several different risk factors. In the fetal period, these include the ultra position of the fetal lower limbs; a breech position of the fetus; left hip joint pressure on the sacrum before and during head delivery; oligohydramnios—intrauterine narrowness; and primigravida.

Risk factors present in the postnatal period (up until the end of the 3rd month of life) include incorrect diapering and extra-articular contractures.

 **(B)** **Late (secondary) developmental deformity of the hip**

This disorder occurs in the postnatal period, after the 3rd month of life. Risk factors include improper care, neurogenic disorders (e.g., disorders of muscular balance in cerebral palsy, myelomeningocele, perinatal neuromuscular dystonia), inflammatory conditions (e.g., viral or bacterial hip inflammation), extra-articular contractures within e.g., adductor muscles of the hip caused by idiopathic muscle fibrosis, ionizing radiation fibrosis, postinflammatory fibrosis (viral muscle damage, bacterial descent processes after abscesses), and fibrosing postpartum hematomas.

**III.**  **Developmental disorders of the hip associated with “packaging problems” and “production problems” (D*mix*H)**

In these disorders, we deal with situations in which the deformity has a secondary effect on tissue quality, or deformity changes are superimposed upon dysplastic changes.

**(1)**  **Developmental dysplasia and deformity of the hip**
  **(A)**  **Early dysplastic disorder with late (secondary) deformity changes**

The dysplastic hip joint with a disturbed congruence is affected later by an additional iatrogenic external mechanical force, e.g., associated with incorrect diapering.

 **(B)** **Early deformity disorder with late (secondary) dysplasia changes**

The developmentally deformed hip joint is later overlaid with the pathomechanism of the production disorder of the dysplasia type.

## 4. Conclusions

Dysplasia, malformation, and deformity are three of the four basic pathomechanisms leading to developmental hip disorders that are often not disambiguated at the clinical level and are therefore incorrectly named. Because malformation is relatively readable for the clinician, it is the least difficult to recognize. However, it is far more difficult to distinguish between dysplasia and deformity, because existing standards do not provide explicit methods along with a full range of diagnostic options. Nevertheless, this distinction is crucial and not merely a theoretical problem—it may influence medical practice, affecting patients’ quality of life and reducing treatment costs.

To achieve this goal, it is necessary to perform a full diagnostic process (physical examinations, imaging tests, genetic tests) in the immediate postnatal period to determine whether we are dealing with any of the following:

- An anatomically normal hip joint with normal growth potential;
- Teratogenic congenital dislocation of the hip with disturbed tissue growth potential in the malformation process (embryonic period);
- A dysplastic hip joint with disturbed growth potential in the prenatal period (fetal period) (cases of early dysplasia);
- An initially anatomically normal hip joint, but with a changed genotype that interferes with the potential for tissue growth in the postnatal period (cases of late dysplasia);
- A deformed hip joint with normal growth potential occurring in the prenatal and postnatal periods (cases of early and late developmental deformities);
- A dysplastic hip joint with external deforming mechanical forces affecting it during the fetal or postnatal period;
- A deformed or dysplastic hip joint that is affected by internal or external mechanical forces (joint discongruity) resulting in secondary degenerative tissue changes.

In addition to routine procedures, specialists will likely have to rely on additional genetic testing to account for the possibility of recurrent residual dysplasia that is refractory to treatment despite a properly managed therapeutic process. In addition, such tests may help quickly explain the specific type of dysplasia and late deformity, preventing the premature termination of diagnosis and treatment, and thereby improving the quality of life of patients. The presented procedure would reduce the percentage of undetected diagnostically and clinically mute developmental dysplasia and clinically mute developmental deformity. Summarizing, precise classification determines appropriate treatment, and genetic testing may aid early detection in ambiguous cases.

## Data Availability

No new data were created or analyzed in this study. Data sharing is not applicable to this article.
